# Antibody–Drug Conjugates in Gastrointestinal Oncology: Clinical Efficacy and Inpatient Toxicity Management

**DOI:** 10.3390/jpm16040195

**Published:** 2026-04-01

**Authors:** Ashish Sharma, Harendra Kumar, Ruchir Paladiya, Rajvardhan Sisodia, Hareesha Rishab Bharadwaj, Islam Mohamed, Saqr Alsakarneh, Umar Hayat, Sneh Sonaiya, Hema Sameera Pinnam, Hassam Ali, Dushyant Singh Dahiya

**Affiliations:** 1 Department of Internal Medicine, Yale New Haven Hospital, New Haven, CT 06510, USA; 2 Mayo Clinic, Rochester, MN 55905, USA; 3 Department of Internal Medicine, University of Connecticut School of Medicine, Hartford, CT 06103, USA; 4 Department of Internal Medicine, Mahatma Gandhi Memorial Medical College, Indore 452001, Madhya Pradesh, India; 5 Department of Internal Medicine, Royal Stoke University Hospital, University Hospitals of North Midlands NHS, Staffordshire, Stoke-on-Trent ST4 6QG, UK; 6 Department of Gastroenterology and Hepatology, University of Missouri, Columbia, MO 65212, USA; 7 Department of Internal Medicine, Geisinger Health System, Wilkes-Barre, PA 18711, USA; 8 Department of Internal Medicine, University of Nevada, Las Vegas, NV 89154, USA; 9 Department of Internal Medicine, Jagadguru Sri Shivarathreeshwara Medical College, Mysuru 570015, Karnataka, India; hspinnam@gmail.com; 10 Department of Gastroenterology, Hepatology and Nutrition, East Carolina University Brody School of Medicine, Greenville, NC 27858, USA; 11 Division of Gastroenterology, Hepatology & Motility, The University of Kansas School of Medicine, Kansas City, KS 66160, USA

**Keywords:** antibody-drug conjugates, gastrointestinal oncology, trastuzumab deruxtecan, interstitial lung disease, HER2-positive gastric cancer, inpatient toxicity, CLDN18.2, TROP2, drug-to-antibody ratio, precision oncology

## Abstract

Antibody–drug conjugates (ADCs) are reshaping the therapeutic approach to advanced gastrointestinal cancers by integrating tumor-specific monoclonal antibodies with potent cytotoxic payloads to improve targeted tumor cell destruction while minimizing systemic exposure. Compared to traditional chemotherapy, trastuzumab deruxtecan has significantly improved objective response rates and overall survival in HER2-positive gastric and gastroesophageal junction tumors after trastuzumab-based therapy. This supports its role as an important second-line or later treatment option. The ongoing advancement of ADCs targeting CLDN18.2, TROP2, and CEACAM5 indicates that this therapeutic category will continue to expand across gastrointestinal neoplasms. Nonetheless, these advancements are accompanied by a specific and clinically significant toxicity profile. Hematologic suppression, gastrointestinal side effects, hepatotoxicity, and notably interstitial lung disease (ILD) are essential consequences that may need inpatient assessment and care. Interstitial lung disease (ILD), although uncommon, may be severe or lethal if not identified immediately and treated swiftly with medication cessation and corticosteroids. In hospitalized patients, distinguishing ADC-related toxicity from infection or disease progression is often difficult owing to overlapping clinical manifestations, requiring meticulous evaluation and interdisciplinary cooperation. As ADCs are integrated into earlier treatment lines and across a broader patient population, hospital systems must evolve to ensure prompt identification, consistent management protocols, and efficient collaboration between oncology and inpatient teams. This study analyzes the mechanisms, clinical effectiveness, and safety profile of ADCs in gastrointestinal oncology, pointing out the importance of institutional preparedness to safely incorporate these medicines into standard clinical practice. These features also align ADC therapy with personalized medicine by emphasizing biomarker-guided patient selection and individualized toxicity monitoring.

## 1. Introduction

Antibody–drug conjugates (ADCs) have swiftly transformed the treatment of advanced gastrointestinal (GI) malignancies by attaching tumor-specific monoclonal antibodies to powerful cytotoxic agents. This is feasible because of dependable linker technology. This technique reduces systemic exposure by delivering cytotoxic payloads selectively to antigen-expressing tumor cells, while membrane-permeable payloads such as DXd can also eliminate adjacent antigen-low or antigen-negative cells through a bystander effect. Trastuzumab deruxtecan (T-DXd), an anti-ERBB2 antibody–drug conjugate linked to a topoisomerase I inhibitor, has shown clinically meaningful enhancements in response and survival rates in HER2-positive advanced gastric and gastro-oesophageal junction malignancies. In the second-line or later setting after trastuzumab-based therapy, T-DXd significantly improved the objective response rate and overall survival compared to physician’s choice chemotherapy in the phase II DESTINY-Gastric01 trial [1]. First-line therapy remains chemotherapy plus trastuzumab, with pembrolizumab added in selected PD-L1-positive disease [2,3]. The global significance of ADC therapy in gastric cancer was validated by the DESTINY-Gastric02 research, which subsequently showed similar results in Western populations. The use of antibody–drug conjugates (ADCs) in gastrointestinal oncology is expected to expand as investigations into CLDN18.2, TROP2, and CEACAM5 progress. This expanding role also reflects a personalized medicine paradigm in which treatment selection depends on tumor antigen profiling and careful patient-specific risk assessment.

Notwithstanding these advancements, ADC treatment has a distinct risk profile compared to conventional chemotherapy, necessitating heightened caution from physicians. Notable adverse effects that may need hospitalization are hematologic toxicities, gastrointestinal complications, and particularly interstitial lung disease (ILD) [4,5]. To guarantee the safety and efficacy of novel therapies in clinical practice, hospital physicians must recognise and address the adverse effects associated with the growing use of antibody–drug conjugates (ADCs) at earlier treatment stages and among larger patient populations.

## 2. Mechanism of Action and Structural Design of Antibody–Drug Conjugates

**Figure 1:**
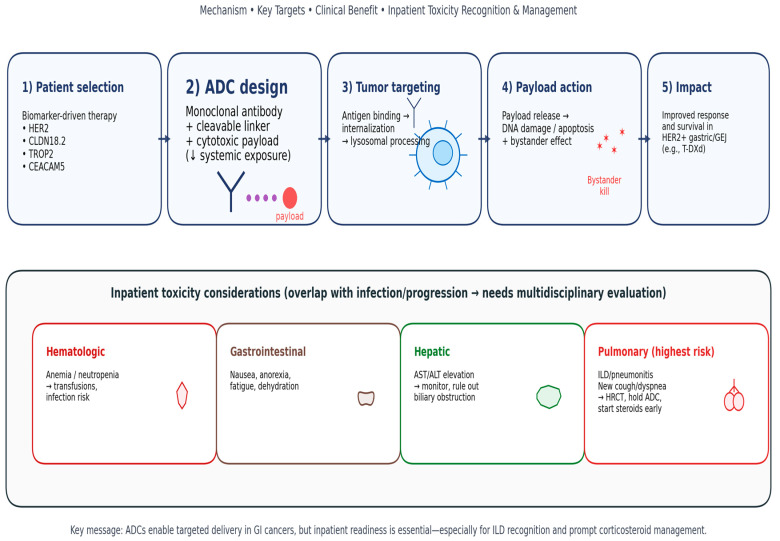
Schematic overview of antibody–drug conjugate (ADC) structural components and intracellular mechanism of action. Upon binding to a tumor-associated antigen, the ADC is internalized, and the cytotoxic payload is released following lysosomal processing. Membrane-permeable payloads (e.g., deruxtecan) can additionally diffuse to neighboring tumor cells, generating a bystander effect.

Antibody–drug conjugates (ADCs) consist of three components: a monoclonal antibody that targets a tumor-associated antigen, a cytotoxic payload, and a chemical linker that connects the antibody to the payload [1,6]. This mechanism facilitates the direct administration of chemotherapy to cancer cells, sparing healthy tissues from damage [4,7]. The antigen interacts with the ADC–receptor complex, prompting endocytosis; the linker is then cleaved by lysosomal enzymes, releasing the cytotoxic payload into the cellular environment. The efficacy of an ADC is contingent upon the number of antigens it can bind its cellular entry efficiency, the stability of its linker, and the potency of its payload (Figure 1).

Trastuzumab deruxtecan (T-DXd) is the most sophisticated amalgamation of an antibody and a therapeutic agent for the treatment of gastric and intestinal cancers. It specifically targets ERBB2 (HER2) and incorporates a cleavable tetrapeptide linker that attaches it to a topoisomerase I inhibitor payload (deruxtecan) [1,5]. T-DXd has a high drug-to-antibody ratio (about 8), yet its linker–payload design allows this high DAR to be maintained with acceptable stability rather than the instability seen with many older high-DAR constructs [5,8,9,10]. Trastuzumab deruxtecan is specifically engineered to have a high drug-to-antibody ratio of around 8, thereby providing a powerful cytotoxic payload that is internalised and selectively cleaved by lysosomal enzymes overexpressed in cancer cells. The membrane-permeable DXd payload, which is largely uncharged at physiologic pH, induces a “bystander effect,” facilitating its dissemination to adjacent tumour cells that lack HER2 expression [11]. The powerful payload is delivered precisely into HER2-overexpressing tumour cells to cause DNA damage, resulting in death of the target tumour cells and adjacent cells via a bystander effect, facilitated by the cytotoxic payload’s membrane permeability.

HER2 expression often exhibits variability in gastric malignancies, making this significant [11,12]. In this setting, the bystander effect is particularly advantageous because membrane-permeable DXd can diffuse from HER2-expressing cells into adjacent HER2-low or HER2-negative tumour cells, an effect that is less pronounced with non-membrane-permeable payloads [11,12]. To achieve an optimal balance between safety and effectiveness, the linker must be stable. Pharmaceuticals pose greater risks when administered prematurely, whereas excessively stable linkers may inhibit drug cellular uptake, leading to reduced targeting of cancer cells and potentially compromising treatment outcomes [4]. Advancements in linker chemistry and payload design have markedly enhanced the therapeutic index. A greater number of antibody–drug conjugates (ADCs) aimed against CLDN18.2, TROP2, and CEACAM5 are under development. It remains crucial to optimise antigen selection, determine the optimal drug-to-antibody ratio, and enhance the payload’s properties to achieve excellent outcomes with minimal toxicity in gastrointestinal malignancies [7].

The drug-to-antibody ratio (DAR) is a crucial factor that affects the efficacy and safety of antibody–drug conjugates (ADCs), influencing pharmacokinetics, biodistribution, and the therapeutic index [13]. Research indicates that the Drug-to-Antibody Ratio (DAR) substantially influences pharmacokinetic characteristics: many earlier conjugates with a DAR exceeding 6 exhibit rapid clearance and heightened hepatic accumulation, whereas T-DXd shows that a DAR of about 8 can be feasible when the linker–payload platform is optimized for stability and controlled release [5,9,10,13]. The elevated DAR of T-DXd, around 8, enhances its efficacy while also affecting its toxicity profile [9,14]. Recent research indicates that optimum DAR may fluctuate depending on target antigen expression, internalisation kinetics, and tumour heterogeneity [9,10].

The pharmacokinetic complexity of ADCs encompasses multiple analytes: intact ADC, unconjugated antibody, and released payload, each with unique pharmacokinetic characteristics [9]. The temporal fluctuations in DAR resulting from in vivo deconjugation influence payload release and therapeutic efficacy [9]. Post-administration, antibody–drug conjugates (ADCs) exist in the body as separate molecular entities, with the drug-to-antibody ratio (DAR) fluctuating over time due to variability in conjugation and in vivo deconjugation rates [15]. This complexity requires advanced bioanalytical techniques and pharmacokinetic modeling to characterise ADC disposal [16] thoroughly.

Novel site-specific conjugation technologies enable the uniform manufacturing of antibody–drug conjugates (ADCs) with consistent drug-to-antibody ratios (DAR), thereby enhancing pharmacokinetic predictability and potentially mitigating toxicity relative to traditional stochastic conjugation techniques [6]. Site-specific ADCs with varying DARs have shown similar pharmacokinetics across all DARs, with dose-dependent effectiveness, indicating that this platform facilitates the empirical identification of the optimal DAR for specific targets [6]. Dosing based on body weight may not be ideal for all antibody–drug conjugates (ADCs) [17], as personal patient factors, such as lean body mass, albumin concentrations, and immune system markers, can significantly influence ADC pharmacokinetics and efficacy. Innovative measures, such as lean body mass, albumin concentrations, and indicators of the innate immune system, especially FcγR expression, have been linked to ADC clearance and distribution volume, suggesting opportunities for precision dosing [18]. These observations support personalized medicine strategies in which dose selection and monitoring are individualized according to patient-specific pharmacokinetic determinants.

## 3. Clinical Efficacy in Gastrointestinal Malignancies

For advanced HER2-positive gastric and gastroesophageal junction (GEJ) tumors, outcomes after progression on trastuzumab-based first-line therapy have historically been poor. Standard second-line chemotherapy response rates are typically below 20%, and the median overall survival is less than 1 year [2,3,4]. Trastuzumab deruxtecan (T-DXd) has made a big difference in this area. In the randomized phase II DESTINY-Gastric01 research, T-DXd exhibited an objective response rate (ORR) of 42.9%, in contrast to 12.5% for physician-selected chemotherapy (irinotecan or paclitaxel) [1]. This led to a significant overall survival advantage, with a median overall survival of 12.5 months compared to 8.4 months (hazard ratio for death, 0.59; 95% CI, 0.39–0.88) and higher progression-free survival (5.6 vs. 3.5 months) [1]. The superiority of T-DXd over a comparator arm that included irinotecan, despite both strategies using topoisomerase I inhibition, underscores the importance of targeted delivery rather than payload class alone [1].

The phase II DESTINY-Gastric02 study confirmed similar outcomes in a Western cohort that had progressed after trastuzumab-based therapy, with a validated overall response rate (ORR) of 41.8%, a median progression-free survival of 5.6 months, and a median overall survival of 12.1 months [6]. These results showed that the benefits may be repeated in other places and with different types of therapy.

Across studies, response rates with T-DXd in HER2-positive gastric cancer remain above 40%, indicating a clinically substantial improvement in a population historically recognized for suboptimal second-line effectiveness [1,19]. These findings position T-DXd as an important HER2-targeted option in the second-line or later setting after trastuzumab-based therapy and provide the groundwork for further exploration of other targets in gastrointestinal cancer.

Multiple resistance mechanisms have been identified for T-DXd and other ADCs in gastrointestinal cancers, with important implications for patient management and the sequencing of therapies [8,20]. Understanding these mechanisms is critical for developing rational combination strategies and selecting subsequent therapies [20]. HER2 expression loss or downregulation occurs in approximately 49% of patients progressing on T-DXd, with 52% of these cases exhibiting complete HER2 loss [21]. This represents a major mechanism limiting long-term efficacy [21]. Spatial profiling studies reveal that one-third of trastuzumab-resistant HER2-positive gastric cancers undergo epithelial–mesenchymal transition (EMT), with upregulation of PD-L1 and CCL2. At the same time, another third activates endoplasmic reticulum-associated degradation (ERAD) pathways [12]. After trastuzumab treatment, some patients have been observed to lose HER2 expression, potentially rendering their disease refractory to subsequent HER2-directed therapy [19]. HER2 mutations in the trastuzumab binding interface, such as V597M and P593R, have also been identified and validated as promoting T-DXd resistance [21].

ATP-binding cassette (ABC) transporters, particularly ABCG2 and ABCB1, remain plausible contributors to drug efflux and resistance, although DXd was designed to limit P-glycoprotein-mediated efflux and the clinical importance of this mechanism in gastric cancer remains under investigation [22]. Pharmacological inhibition of these transporters or use of alternative payloads, such as eribulin in BB-1701, may overcome this resistance mechanism [22]. Prior irinotecan therapy, a topoisomerase I inhibitor, has been associated with worse outcomes with T-DXd (HR 4.91 for progression), but it remains uncertain whether this reflects payload cross-resistance, more aggressive disease biology, or both [23]. Alterations in receptor internalization, lysosomal processing, and intracellular trafficking can prevent effective payload release even when target antigen expression is maintained [24]. Decreases in HER2 expression correspond to reductions in T-DXd internalization and to major increases in the drug’s IC50 for tumor growth inhibition [21].

An immunosuppressive microenvironment, HLA loss, and increased oxidative phosphorylation pathways have been observed in T-DXd-resistant gastric cancers [12]. The tumor microenvironment plays a critical role in limiting ADC delivery and efficacy [25]. As a strategy to overcome impaired T-DXd binding and internalization, low-dose combinations of T-DXd with TROP2-directed ADCs have been tested and found to more uniformly deliver T-DXd payloads, thereby overcoming resistance mediated by HER2 loss [21].

## 4. Toxicity Burden of Antibody–Drug Conjugates

Therapeutic advantage, which refers to the benefits of a treatment, is associated with clinically relevant harm. In ADC investigations, more than 50–60% of patients had grade 3 or higher adverse events, but these aggregate rates include both laboratory abnormalities and clinically significant toxicities that require intervention [4,5]. Hematologic laboratory abnormalities are common; in DESTINY-Gastric01, grade 3/4 decreased neutrophil count and anemia occurred in approximately 51% and 38% of patients, respectively [1]. Patients receiving ADC therapy often possess an extensive history of prior treatments and diminished marrow reserve, increasing their vulnerability to infections, requiring transfusions, and extending recovery periods.

Therapeutic modifications and dosage interruptions are additional indicators of the clinical burden of toxicity. In investigations of trastuzumab deruxtecan for gastrointestinal malignancies, 20–30% of patients required dose modifications, and 10–15% had to discontinue treatment due to adverse effects [5,8]. In DESTINY-Gastric01, 15% of patients discontinued medication owing to adverse events [1]. Non-hematologic toxicities, including nausea, fatigue, anorexia, and gastrointestinal symptoms, are also common and may contribute to dehydration, functional impairment, and unanticipated hospitalizations, particularly in those who are already frail or have advanced metastatic disease [1,4,5]. The findings indicate that ADC-related toxicity transcends outpatient therapy and is essential for inpatient care, as it requires close monitoring and management of symptoms to prevent complications such as dehydration and functional impairment.

## 5. Interstitial Lung Disease: A Critical Inpatient Risk

Interstitial lung disease (ILD) remains the most severe result. In DESTINY-Gastric01, 9.6% of patients experienced drug-induced interstitial lung disease (ILD), with some cases leading to fatalities [1]. Extensive studies, including anti-ERBB2 ADCs, indicate that interstitial lung disease (ILD) occurs in 8% to 15% of people, with mortality directly linked to delayed diagnosis or late commencement of corticosteroids [5]. The onset of symptoms may need many months, hence reducing the likelihood that physicians would first identify any underlying issues. Initial symptoms are often ambiguous, including mild dyspnea, cough, or diminished exercise tolerance; nonetheless, the illness may progress rapidly. Inpatient teams often face similar situations due to the lack of set institutional standards for assessment and treatment.

The severity of ILD is classified according to the Common Terminology Criteria for Adverse Events (CTCAE), ranging from grade 1 (asymptomatic) to grade 5 (fatality) [26,27]. The typical duration to ILD development is around 4 months, though it may range from a few days to over a year, requiring diligent long-term surveillance [28]. Empirical evidence suggests that the incidence of ILD associated with T-DXd therapy ranges from 2.7% to over 15%, depending on tumour type and patient demographics [28,29].

Weekly clinical assessments, including medical history, physical examination, and pulse oximetry, are advised for grade 1 interstitial lung disease [26]. Radiologic and pulmonary assessments, including pulmonary function tests, should be repeated after 1–2 weeks or as clinically warranted [26]. For grade 2 incidents, clinical and radiological evaluations should be conducted every 2 to 3 days [26]. Grade 3–4 incidents need hospitalisation with suitable, intense monitoring [26]. In clinically stable patients with suspected ILD and a credible infectious differential, bronchoscopy with bronchoalveolar lavage should be strongly considered because it remains the diagnostic gold standard for distinguishing drug toxicity from infection before high-dose immunosuppression [30,31]. Almost all doctors report assessing ILD/P after T-DXd initiation using physical examination, a symptom checklist, and pulse oximetry at each visit. Conversely, lung CT scans, echocardiograms, chest X-rays, or pulmonary function tests are performed less frequently [29].

In asymptomatic instances (grade 1), oral corticosteroids (prednisolone 0.5 mg/kg/day or similar) should be commenced and tapered slowly, generally over 6–8 weeks, after clinical and radiographic improvement [26,27,30]. T-DXd should be interrupted, and rechallenge may be considered only after complete resolution to grade 0 following careful multidisciplinary review; rapid steroid tapering increases recurrence risk [26,30,32]. A pooled study including 2145 patients from nine clinical studies revealed that 23.3% of patients who recovered from grade 1 ILD were retreated with T-DXd, with a median retreatment duration of 85 days [32].

For mild symptomatic cases (grade 2), T-DXd should be permanently discontinued and systemic corticosteroids should be started promptly at a dose of 1 mg/kg/day of prednisolone equivalent for 2–4 weeks, followed by a slow taper that usually extends 6–8 weeks [26,27,30]. If no improvement is seen after 3–5 days, the dosage may be increased to 2 mg/kg/day and/or transitioned to intravenous delivery [26]. Hospitalization is recommended with a low threshold and rechallenge should not be attempted after grade 2 ILD [26,30].

In severe instances (grades 3–4), hospitalization is essential, commencing with pulse treatment with methylprednisolone at 0.5–1 g/day for a minimum of 3 days, succeeded by prednisolone at 1.0 mg/kg/day for 2–4 weeks, with a gradual tapering that generally extends 6–8 weeks [26,27,30]. Oxygen supplementation, broad-spectrum antibiotics, and supportive therapies (calcium, vitamin D, proton pump inhibitors, and antihyperglycemics) are important [26]. In cases where an underlying infection cannot be excluded or to prevent subsequent infections, the use of broad-spectrum antibiotics may be warranted [26,31]. In corticosteroid-refractory cases, additional immunosuppressive drugs such as mycophenolate mofetil or cyclophosphamide are more commonly recommended; infliximab should be used cautiously, and intravenous immunoglobulin may also be considered in selected cases [26,30,31]. Empirical evidence suggests that treatment strategies often diverge from prescribed information, underscoring the need for improved physician education and institutional guidelines [29,33].

## 6. Diagnostic Complexity in Hospitalized Patients

The constitutional and gastrointestinal effects complicate the assessment process. Persistent nausea, anorexia, and fatigue may indicate disease progression and might be linked to the topoisomerase I inhibitor component of T-DXd. Elevated hepatic enzymes, often mild to moderate, may be erroneously interpreted as tumor-induced liver damage in people with metastatic involvement; ADC-related injury more often presents with a transaminase-predominant pattern and, in some cases, sinusoidal obstruction syndrome, whereas tumor progression or biliary obstruction more often produces a cholestatic pattern [5]. Clinical worsening often signifies several underlying causes, necessitating a collaborative multidisciplinary assessment.

This uncertainty often affects the total length of hospitalization. Decisions about imaging, antibiotic treatment, transfusion support, and the escalation of care are sometimes undertaken before the full diagnosis of the fundamental cause of deterioration [8,34,35]. A patient exhibiting increased fatigue and reduced oral intake may undergo a thorough evaluation, which may eventually suggest cumulative therapy toxicity rather than advancing malignancy. Conversely, hastily attributing symptoms to medication may lead to overlooking an underlying illness or a progressive disease [12,20,36]. It is essential to assess treatment regimens and test trends thoroughly, and to involve oncology specialists from the beginning. The first 24 to 48 h of reevaluation often ascertain whether the therapy is enough or excessive.

Precise biomarker assessment is crucial for better patient selection and treatment efficacy with ADCs in gastrointestinal cancers [2,37]. The significant diversity in gastric cancer indicates that it requires thorough biomarker profiling and the evaluation of geographic and temporal fluctuations in antigen expression [37]. HER2 status must be evaluated by immunohistochemistry (IHC), with in situ hybridization (ISH) used for ambiguous cases [3]. HER2-positive gastric cancer is characterized by IHC 3+ or IHC 2+/FISH+ results [3]. The NCCN Guidelines Panel advises that HER2 (ERBB2) IHC should be conducted first, followed by ISH procedures in instances exhibiting 2+ (equivocal) expression by IHC [3]. Positive (3+) or negative (0 or 1+) HER2 IHC findings do not need further ISH testing [3]. Cases exhibiting HER2 (ERBB2) with a CEP17 ratio ≥ 2 or an average HER2 (ERBB2) copy number ≥ 6.0 signals/cell are classified as positive by ISH/FISH [3]. This biomarker-driven framework is central to personalized medicine, because ADC selection depends on accurate and sometimes repeated characterization of target expression.

Gastric malignancies have significant intratumoral HER2 heterogeneity, surpassing that seen in breast cancer [37]. Patients with variable HER2 expression often have reduced progression-free survival with trastuzumab-containing regimens, in contrast to those with homogeneous HER2 expression [37]. This heterogeneity also explains the relevance of the bystander effect, because membrane-permeable payloads such as DXd can diffuse into adjacent HER2-low or HER2-negative tumor cells more effectively than non-permeable payloads [11,12,37]. Discrepant HER2 expression between primary tumors and metastatic lesions is observed in a significant fraction, highlighting the importance of timing and anatomical site for molecular evaluation [37]. In people with advanced or metastatic gastric adenocarcinoma, repeat biomarker testing may be warranted upon clinical or radiologic progression [3].

CLDN18.2 positivity is characterized by ≥75% of live tumor cells exhibiting moderate to strong (2+ or 3+) membrane staining by immunohistochemistry for zolbetuximab treatment [2,3]. This threshold for a naked monoclonal antibody should not be assumed to apply directly to CLDN18.2-targeted ADCs. In the development of CLDN18.2-targeted antibody–drug conjugates (ADCs), positive criteria range from ≥40% to ≥75% with a minimum intensity of ≥2+, and the bystander effect of membrane-permeable payloads may allow activity at lower or more heterogeneous expression levels than those required for zolbetuximab [38]. CLDN18.2 is expressed in 21–95% of stomach or gastroesophageal junction adenocarcinomas, with 31–86% exhibiting moderate-to-high levels of CLDN18.2 expression [38]. The VENTANA CLDN18 (43-14A) RxDx Assay has shown strong, consistent analytical performance and clinical applicability as a companion diagnostic for first-line zolbetuximab in conjunction with chemotherapy [39].

It is advisable to test for microsatellite instability (MSI) or mismatch repair (MMR) status and PD-L1 expression in all newly diagnosed gastric cancer cases to inform therapeutic decisions [3,37]. Novel biomarkers, such as TROP2, CEACAM5, and HER3, are being explored as targets for antibody–drug conjugates (ADCs) [20]. Currently, many targeted therapy drugs have received FDA approval for the treatment of gastric cancer, with IHC/ISH/targeted gene PCR being the favored method for initial biomarker evaluation [3]. In situations when tissue is scarce or a biopsy is impractical, multigene panel testing of ctDNA via CLIA-certified labs may reveal targetable mutations. Nevertheless, unfavorable outcomes must be analyzed with prudence [3]. However, ctDNA cannot assess protein expression or intratumoral heterogeneity, so repeat tissue biopsy remains the gold standard at progression when feasible [3,37]. The identification of mutations, changes, or gene fusions in DNA derived from gastric carcinomas may reveal targetable alterations or the emergence of clones with altered treatment response characteristics [3].

## 7. System-Level Impact and the Need for Institutional Adaptation

ADC-associated problems impose a considerable strain on systems [12]. Patients experiencing hypoxia or neutropenic fever require advanced imaging, a subspecialist consultation, and meticulous monitoring [40]. In cases of suspected interstitial lung disease (ILD), a high-resolution CT scan, bronchoscopy with bronchoalveolar lavage, or supplementary respiratory assistance may be necessary. Corticosteroid therapy presents additional risks, including hyperglycemia, infection, and functional impairment. As antibody–drug conjugates (ADCs) are used in earlier therapy settings, even minor toxicity can lead to increased hospital visits and greater resource need, particularly for managing adverse effects and ensuring patient safety during treatment.

A robust biological justification underpins the integration of antibody–drug conjugates with immunotherapy [41,42]. ADCs elicit immunogenic cell death (ICD), which releases tumor antigens and damage-associated molecular patterns that activate dendritic cells and enhance T-cell infiltration into the tumor microenvironment [41,43]. Topoisomerase I inhibitor payloads, including DXd, appear particularly well suited to induce ICD, providing a mechanistic rationale for ADC–immunotherapy combinations [41,43]. This successfully converts immunologically “cold” cancers into “hot” tumors, fostering synergy with immune checkpoint drugs that rejuvenate fatigued T cells [41,42].

Preclinical studies indicate that antibody–drug conjugates (ADCs) containing pyrrolobenzodiazepine (PBD) or tubulysin payloads elicit immune responses that inhibit tumor development following rechallenge, exhibiting enhanced anticancer efficacy in immunocompetent compared to immunodeficient animals [44]. The depletion of CD8+ T cells nullifies ADC activity, hence affirming the essential function of adaptive immunity [44]. T-DM1 enhances tumor-infiltrating lymphocytes in human breast malignancies and murine models, and its combination with PD-1/CTLA-4 inhibition demonstrates superior effectiveness compared to either drug individually [45,46].

Numerous ADC–immunotherapy combinations are being explored in gastric cancer [47,48]. The GEMINI-Gastric study (NCT05702229) is assessing innovative combinations of AZD0901 (a CLDN18.2-targeted antibody–drug conjugate) with immunotherapeutic drugs in HER2-negative advanced gastric/gastroesophageal junction cancer [47]. Early-phase studies integrating T-DXd with pembrolizumab or nivolumab are now underway across many tumor types [41,48]. A comprehensive review and meta-analysis of antibody–drug conjugate and immune checkpoint inhibitor combinations in solid tumors revealed that 55.3% of patients had grade ≥ 3 adverse events, with an overall response rate of 48.8% and a complete response rate of 9.0% [49]. These regimens also require particular caution for pneumonitis, because both ADCs such as T-DXd and immune checkpoint inhibitors can independently cause ILD, complicating diagnosis and management [41,48,49].

Principal issues include establishing appropriate dosage (potentially using lower ADC levels to reduce toxicity while preserving synergy), addressing overlapping toxicities, particularly pneumonitis/ILD, and developing predictive biomarkers for combination efficacy [41,48,49]. Innovative ADC architectures, such as bispecific ADCs, immune-stimulating ADCs, and dual-payload designs, are being explored to augment therapeutic synergies [41,48]. Combinations of antibody–drug conjugates (ADCs) with immuno-oncology agents, such as PD-1 or PD-L1 antibodies, OX40 ligand, or GITR ligand fusion proteins, have shown synergistic antitumour responses in preclinical animals [44].

## 8. Emerging Targets and Future Directions

**Figure 2:**
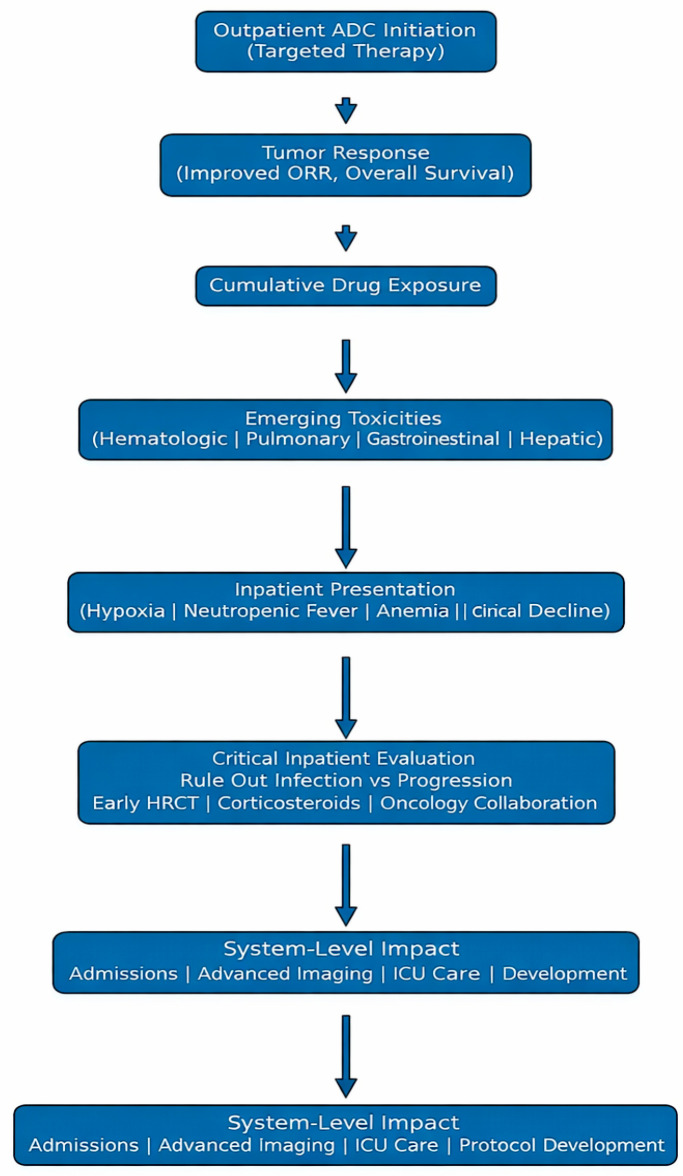
Clinical trajectory of antibody–drug conjugate therapy and key points of inpatient risk. The diagram illustrates the phases of ADC treatment from initial administration through disease response or progression, highlighting the specific time windows and clinical scenarios in which toxicity-related hospitalizations are most likely to occur. ILD: interstitial lung disease; T-DXd: trastuzumab deruxtecan.

The development pipeline signifies more expansion. Antibody–drug conjugates (ADCs) targeting CLDN18.2, TROP2, and CEACAM5 are advancing in the treatment of gastrointestinal malignancies [22,50]. An increase in the applications of these medications will result in more exposure for people during hospital stays. Algorithms for institutional toxicity are less sophisticated than those developed for immune checkpoint inhibitors, which may lead to underestimating the risks associated with ADCs. Although CLDN18.2-, TROP2-, and CEACAM5-directed ADCs share structural principles, their toxicity profiles are not interchangeable; the pulmonary toxicity signal is best established with T-DXd and should not be generalized uniformly to the entire class [22,50]. ADC toxicities display similarities to cytotoxic chemotherapy and targeted therapies. Still, agent-specific pulmonary vigilance is needed, particularly with T-DXd, because delayed recognition can lead to severe respiratory complications. The enhancements in survival rates for HER2-positive gastric cancer validate the therapeutic effectiveness of ADC treatment [51,52]. The effectiveness of institutional adaptation will determine the safety of implementation. Early identification of interstitial lung disease (ILD), consistent use of corticosteroids, and effective communication between hospital medicine and oncology are essential. Hospital staff should receive regular training on the toxicities of antibody–drug conjugates (ADCs). For precision oncology to be effective, the identification and management of toxicity must be equally precise. As antibody–drug conjugates (ADCs) transform expectations for gastrointestinal cancer therapies, healthcare systems must respond with equal gravity by implementing comprehensive staff training programs to ensure they are well-equipped to manage the associated toxicities effectively. Clinical innovation has its greatest impact when patient preparedness in the hospital aligns with advances in therapies. Table 1 summarizes key clinically relevant toxicities, their occurrence, hospital presentation, and therapeutic concerns. Figure 2 depicts the clinical progression of ADC treatment and significant inpatient vulnerabilities.

## 9. Conclusions

Antibody–drug conjugates have fundamentally changed the treatment of advanced gastrointestinal malignancies, offering patients with limited second-line options a meaningful chance at improved survival. The clinical impact of T-DXd in HER2-positive gastric cancer and the emergence of CLDN18.2-targeted therapies illustrate the transformative potential of this drug class [2,37,53].

However, superior outcomes bring with them greater clinical responsibility. ADC-related toxicities like hematologic suppression, pulmonary complications, and GI adverse effects can begin as manageable Grade 1–2 events and escalate rapidly if not recognized and addressed. In the inpatient setting, distinguishing drug toxicity from infection or disease progression demands structured clinical assessment and close oncology involvement. Multidisciplinary collaboration is not incidental to safe ADC therapy; it is integral to it.

Realizing the full potential of ADC-based therapy in GI oncology will require simultaneous progress across several domains: developing rational combination strategies to overcome resistance mechanisms, refining patient selection through comprehensive biomarker profiling, standardizing institutional protocols for ADC toxicity recognition and management, and establishing evidence-based guidelines for ADC combinations with immunotherapy and other systemic agents [20,37,41,48]. Together, these steps will help embed ADC therapy within a more complete personalized medicine model in GI oncology.

As these agents are administered to broader patient populations and at earlier disease stages, inpatient teams will encounter ADC-related toxicities with increasing frequency. Building the knowledge and infrastructure to manage these presentations safely, through physician education, clear institutional protocols, and effective oncology–hospitalist communication, it is an investment that will determine the real-world safety of ADC therapy. Clinical innovation in oncology reaches its greatest impact when advances in therapy are matched by equal advances in the capacity to deliver that therapy safely [31,33].

## Figures and Tables

**Table 1:** Clinically Significant Toxicities of Antibody–Drug Conjugates in Gastrointestinal Malignancies: Inpatient Recognition and Management.

Toxicity	Reported Incidence (Grade ≥ 3 When Available)	Typical Time to Onset	Key Inpatient Presentation	Immediate Evaluation Priorities	Management Principles	Key Refs.
Interstitial Lung Disease (ILD)	8–15% overall; 9.6% in DESTINY-Gastric01 (including fatal cases)	Median ~4–6 months, but variable	New hypoxia, dry cough, exertional dyspnea, subtle oxygen decline	High-resolution CT chest; exclude infection; pulmonary consultation; consider bronchoscopy with bronchoalveolar lavage	Interrupt drug; grade 1 corticosteroids with slow taper and consider rechallenge only after complete resolution; permanently discontinue for grade ≥ 2	[1,26,30]
Anemia	Approximately 38% Grade 3/4 in DESTINY-Gastric01	Often cumulative over cycles	Fatigue, dyspnea, symptomatic anemia, transfusion need	CBC with differential; assess bleeding vs. marrow suppression	Transfusion support; consider dose modification; coordinate with oncology	[1]
Neutropenia	Approximately 51% Grade 3/4 in DESTINY-Gastric01	Early cycles or cumulative	Fever, infection, neutropenic sepsis	CBC; blood cultures; infection workup	Broad-spectrum antibiotics; G-CSF support when indicated; treatment delay or dose reduction	[1]
Nausea/Anorexia	Standard; variable high-grade rates	Early and cumulative	Persistent nausea, weight loss, dehydration	Assess oral intake; electrolytes; rule out obstruction or progression	Aggressive antiemetic regimen; IV fluids; nutritional support; oncology reassessment	[5]
Hepatotoxicity (Transaminase Elevation)	Typically mild–moderate; Grade ≥ 3 less common	Variable	Elevated AST/ALT; sometimes sinusoidal obstruction syndrome	LFT panel; bilirubin/alkaline phosphatase pattern; assess for progression or biliary obstruction	Monitor closely; hold therapy for significant elevation; distinguish transaminase-predominant injury from cholestatic progression	[5]

## Data Availability

The original contributions presented in this study are included in the article. Further inquiries can be directed to the corresponding author.

## Abbreviations

The following abbreviations are used in this manuscript:

Abbreviation	Full Term
ABC	ATP-binding cassette
ADC(s)	Antibody–drug conjugate(s)
ALT	Alanine aminotransferase
AST	Aspartate aminotransferase
CBC	Complete blood count
CEACAM5	Carcinoembryonic antigen-related cell adhesion molecule 5
CLDN18.2	Claudin 18.2
CLIA	Clinical Laboratory Improvement Amendments
CT	Computed tomography
ctDNA	Circulating tumour DNA
CTCAE	Common Terminology Criteria for Adverse Events
DAR	Drug-to-antibody ratio
ERAD	Endoplasmic reticulum-associated degradation
EMT	Epithelial–mesenchymal transition
FDA	Food and Drug Administration
FISH	Fluorescence in situ hybridisation
G-CSF	Granulocyte colony-stimulating factor
GEJ	Gastroesophageal junction
GI	Gastrointestinal
HER2	Human epidermal growth factor receptor 2 (ERBB2)
HRCT	High-resolution computed tomography
ICD	Immunogenic cell death
IHC	Immunohistochemistry
ILD	Interstitial lung disease
ISH	In situ hybridisation
IV	Intravenous
IVIg	Intravenous immunoglobulin
LFT	Liver function test
MMR	Mismatch repair
MSI	Microsatellite instability
NCCN	National Comprehensive Cancer Network
ORR	Objective response rate
OS	Overall survival
PBD	Pyrrolobenzodiazepine
PD-L1	Programmed death-ligand 1
PFS	Progression-free survival
T-DXd	Trastuzumab deruxtecan
TROP2	Trophoblast cell surface antigen 2
